# Differential Responses of Bilberry (*Vaccinium myrtillus*) Phenology and Density to a Changing Environment: A Study from Western Carpathians

**DOI:** 10.3390/plants13172406

**Published:** 2024-08-28

**Authors:** Martin Kubov, Peter Fleischer, Jakub Tomes, Mohammad Mukarram, Rastislav Janík, Benson Turyasingura, Peter Fleischer, Branislav Schieber

**Affiliations:** 1 Faculty of Forestry, Technical University in Zvolen, 960 01 Zvolen, Slovakia; 2 Institute of Forest Ecology, Slovak Academy of Sciences, 960 01 Zvolen, Slovakia; 3 Administration of Tatra National Park, Tatranská Lomnica, 059 60 Vysoké Tatry, Slovakia; 4 Department of Plant Biology, Universidad de la República, Av. Gral. Eugenio Garzón 780, Montevideo 12900, Uruguay; 5 Department of Environment and Natural Resources, Kabale University, Kabale P.O. Box 317, Uganda; 6 African Center of Excellence for Climate Smart Agriculture and Biodiversity Conservation, Haramaya University, Dire Dawa P.O. Box 138, Ethiopia

**Keywords:** bilberry (*Vaccinium myrtillus* L.), phenology, altitudinal gradient, natural spruce forest, disturbed forest

## Abstract

Environmental factors regulate the regeneration of mountain spruce forests, with drought, wind, and bark beetles causing the maximum damage. How these factors minimise spruce regeneration is still poorly understood. We conducted this study to investigate how the phenology and population dynamics of bilberry (*Vaccinium myrtillus* L.), a dominant understory species of mountain spruce forests, are related to selected environmental factors that are modified by natural disturbances (bark beetle and wind). For this, we analysed bilberry at different sites affected by bark beetles and adjacent undisturbed forests in the Tatra National Park (TANAP) during the growing season (April–September) in 2016–2021, six years after the initial bark beetle attack. The observations were taken along an altitudinal gradient (1100–1250–1400 m a.s.l.) in two habitats (disturbed spruce forest—D, undisturbed spruce forest—U). We found that habitat and altitude influenced the onset of selected phenological phases, such as the earliest onset at low altitudes (1100 m a.s.l.) in disturbed forest stands and the latest at high altitudes (1400 m a.s.l.) in undisturbed stands. Although there were non-significant differences between habitats and altitudes, likely due to local climate conditions and the absence of a tree layer, these findings suggest that bilberry can partially thrive in disturbed forest stands. Despite temperature fluctuations during early spring, the longer growing season benefits its growth.

## 1. Introduction

Climate change influences forest ecosystems worldwide; central European mountain spruce forests are no exception. The vulnerability of mountain Norway spruce (*Picea abies* L. Karst.) forests to wind and bark beetle (*Ips typographus* L.) damage has increased [1,2]. Some studies report that wind disturbances and increasing drought trigger large-scale bark beetle outbreaks [1,3,4]. However, over the past few decades, these infestation events have increased, which poses a challenge to spruce forests in maintaining their resistance and resilience [5,6]. This contributed to the decline of mountain spruce forests in large areas [7]. These changes can also trigger successional processes, leading to changes in vegetation composition and structure [8]. In the first years after wind disturbance in mountain spruce forests, herb species diversity is low because few weedy species occur predominantly. They colonise the surrounding space quickly and reach maturity [7]. However, increased ground-level light exposure, along with enhanced nutrient and water availability, favours the emergence of small-seeded species, i.e., birch and rowan [9,10]. These pioneer tree species create the preparatory stands (the initial stage of stand development) necessary for regeneration after disturbances. In general, pioneer tree species have a larger population at the beginning of forest regeneration but lose significant individuals over the subsequent years. Conversely, climax species, being shade-tolerant species, grow under pioneer tree species [11]. These localities have more water and nutrients and less light and warmth, which are key elements for the growth and survival of climax species [12]. The natural regeneration processes and their dynamics with environmental changes regulate forest stability [13]. Successful regeneration depends on the density and composition of the forest floor. The open forest (disturbed area) has more light and is more nutritious than forest stands with a close canopy [14]. These conditions may disadvantage bilberry (*Vaccinium myrtillus* L.) in competition with other species.

Bilberry is a perennial shrub belonging to the *Ericaceae* family. It is a dominant understory herb species in mountain spruce forests in central Europe [15,16]. In mountainous areas, it is an important ecological component of the forest because it influences the dynamics of the forest, such as soil nutrient and carbon cycles, as well as the regeneration of seedlings [17,18]. Bilberry favours closed forest stands over gaps created by disturbance [15] or sparse forest strands. Large-scale disturbances in mountain spruce forests suppress bilberry cover because the competitive ability of bilberry is limited by understory plants, such as *Rubus idaeus*, *Epilobium angustifolium*, and other plant species with relatively high requirements for sufficient light [15,19]. When a forest regenerates, it gradually increases bilberry abundance, which a high level of underground biomass may also promote [20,21]. On the other hand, bilberry is an important food resource for many animals, such as bears and grouses [22,23]. Bilberry is insect-pollinated but can also self-pollinate, permitting reproduction in habitats where pollinators are scarce. Large-scale disturbances and gap dynamics change microclimate in vegetation composition, influencing bilberry abundance, distribution, and phenological timeline, including flowering and pollination [24,25]. Thus, we were interested in knowing bilberry’s phenology regarding temperature and precipitation along a natural elevation gradient. The elevation gradient is a useful marker for plant responses to external stimuli. Elevation can affect phenology, morphology, physiology, and nutrient status, considering varying air pressure and temperature, wind speed, UV exposure, and soil biochemistry at different heights [24,26]. Differences in spruce forest disturbances are pronounced if they have been destroyed along an elevation gradient. Disturbed forests have higher daytime shortwave radiation, temperature, and lower humidity than undisturbed forests [27,28]. Further, the soil is nutrient-rich from tree uprooting and biomass decomposition [14]. Therefore, more heliophilous, eutrophic, and nitrophilic understory species (such as *Athyrium distentifolium*, *Epilobium angustifolium*, and *Rubus idaeus*) than oligotrophic and acidophilic herb species have been found in the herb cover post-disturbance. Yet little is known about the abundance and phenology of bilberry in mountain spruce forests affected by natural disturbance, specifically bark beetle infestation. Only a few studies in Slovakia have focused on the nutrient status and phenolic compounds in bilberry in different habitats [29,30,31,32]. Still, the literature is limited on vegetation succession after natural disturbance [33,34,35]. Many studies have identified the importance of bilberry to the natural regeneration of forest stands [36,37] but they do not include parts of the Slovak Carpathians. Our work fills this gap and aims to describe the differences in abundance and phenology of bilberry between different habitats (disturbed and undisturbed) at altitudes (1100–1400 m a.s.l.) in the protected areas of the High Tatras National Park. We addressed the following specific questions: (i) Is there any effect of the altitude and (un)disturbed habitat on bilberry’s abundance (density)? (ii) Is there a difference in the interannual dynamics of bilberry phenological events along the elevation gradient and in different habitats?

## 2. Materials and Methods 

### 2.1. Study Area

**Figure 1:**
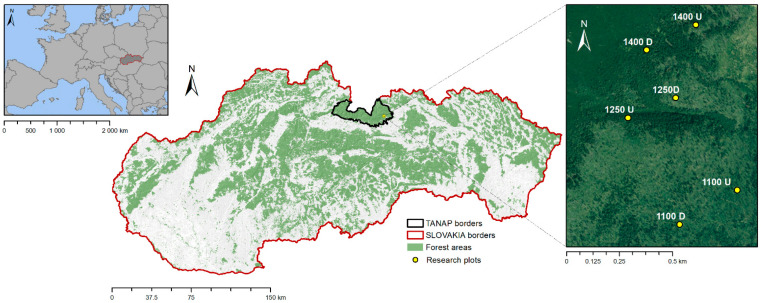
Location of the study area in Europe and in the Slovak Republic (TANAP-Tatra National Park; 1100, 1250, 1400-altitude of research plots (m a.s.l.); D-disturbed forest stand; U-undisturbed forest stand).

The study area is located in the territory of Tatra National Park (TANAP; Figure 1), which is part of the international long-term ecological monitoring and research projects (LTER; EXPEER) [38]. The study sites were on the boundary between undisturbed forests and disturbed forests (bark beetle-infested sites) along an elevation gradient ranging from 1100 to 1400 m a.s.l. More detailed information describing the study area can be found in our previous studies [32,39,40]. The study area represents a type of larch-spruce forest (*Lariceto-Piceetum* community), which is created by Norway spruce (*Picea abies* (L.) Karst.), European larch (*Larix decidua* Mill.), European silver fir (*Abies alba* Mill.), Stone pine (*Pinus cembra* L.), and a few broad-leaved tree species (*Sorbus aucuparia* L., *Salix caprea* L., *Betula* sp.). The vegetation cover is created as patches of *Vaccinio myrtilli-Piceetum* and union *Vaccinio-Piceion*, with permanent elements such as *Vaccinium myrtillus*, *Oxalis acetosella*, *Rubus hirtus*, *Prenanthes purpurea*, *Polygonatum verticillatum*, and *Dryopteris dilatata*. The forest type was classified according to geobiocenosis in a sense, and the names of plant taxa were given accordingly [41].

### 2.2. Sampling Design

The research was conducted during 2016–2021 on undisturbed and disturbed spruce forests by bark beetles. Three parallel research areas (undisturbed vs. disturbed) were selected along a vertical transect (1100–1250–1400 m a.s.l.) in February 2014. The established research area was square, with an area of 900 m^2^ (30 × 30 m). Elevation, slope, exposure, age, and stocking were recorded on each plot in 2016 (Table 1). For the research on undisturbed forest stands, an unmanaged stand with an age of 100–165 years and a stocking density of 0.4–0.8, consisting exclusively of Norway spruce trees, was chosen. However, in May 2014, a part of the undisturbed plot at 1250 m a.s.l. was affected by wind. The wind intervention resulted in a 0.4 reduction in tree density (to stocking 0.4). The forest stand was left unmanaged, and fallen trees were left for self-recovery in this part. On the other hand, bark beetle outbreaks represent disturbed forest stands with standing dead trees.

**Figure 2:**
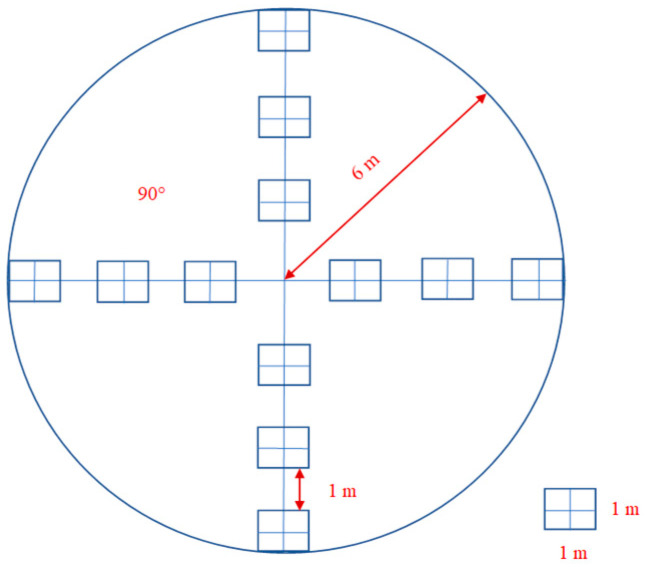
Example of one cluster plot design used in the study comprising 12 square subplots.

The research plots inside the study areas (900 m^2^) were placed using 3 cluster arrangements, as shown in Figure 2. Each cluster includes twelve square (0.25 m^2^) sample plots. The centre of clusters was always distributed within a centre of stands—undisturbed spruce forests or disturbed forests. The plots were placed 6 m from the centre in the north, south, east, and west directions. The plot centre was marked with a metal rod. A small plot cluster can estimate the density of bilberry accurately. The density of bilberry shoots was measured in the same small plot cluster in each research plot in 2016 and 2021.

### 2.3. Climate Data

**Figure 3:**
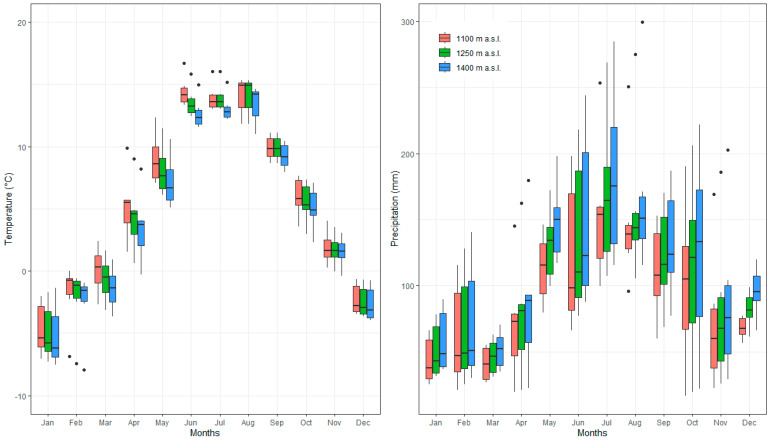
Climate graphs for the three study sites for 2016-2021. (Boxplots of median values of temperature and precipitation and corresponding quartiles. Dots in some boxplots represent potential outliers).

Air temperature and precipitation during the study period (2016–2021) were measured in openings located at three different altitudes (1100–1250–1400 m a.s.l.) using a meteorological station with Minikin Tie and Eri sensors (EMS, Brno, Czech Republic) and a built-in datalogger (Figure 3). However, some of them were broken during the period of the study. Specifically, dataloggers on air temperature in disturbed forest stands at 1250 m a.s.l. during the growing season in 2017 and dataloggers on air temperature in undisturbed forest stands during the growing season in 2019. For this reason, the interpolation method was used to complete the missing data. So, we used the data from the two closest professional meteorological stations, Tatranská Lomnica (830 m a.s.l.) and Skalnaté Pleso (1754 m a.s.l.). Both stations, managed by the Slovak Hydrometeorological Institute (SHMI), were located ˂ 1 km from our study area.

The mean annual air temperature ranged from 5.9 °C at 1100 m a.s.l. to 5.5 °C and 5 °C at 1250 m a.s.l. and 1400 m a.s.l., respectively. The mean air temperature during the growing season (from April to September) was 11.9 °C at 1100 m a.s.l., while higher stations were slightly cooler (10.6 °C at 1250 m a.s.l. and 10.7 °C at 1400 m a.s.l.). The mean annual air temperature of the coldest year (2021) was 4.8 °C (1100 m a.s.l.), 4.2 °C (1250 m a.s.l.), and 3.6 °C (1400 m a.s.l.). On the other hand, one of the warmest years was 2019, when the mean temperature moved from 6.2 °C (1100 m a.s.l.) to 5.1 °C (1400 m a.s.l.) (Table 2). The amount of precipitation in the growing season during the analysed period was 722 mm (1100 m a.s.l.), 797 mm (1250 m a.s.l.), and 871 mm (1400 m a.s.l.). The highest amount was observed in 2017 (from 824 mm in 1100 m a.s.l. to 975 mm in 1400 m a.s.l.), while the least amount of precipitation was found in 2019, from 648 mm (1100 m a.s.l.) to 780 mm (1400 m a.s.l.).

### 2.4. Phenological Observations

Phenological observations were based on the methodology of the Slovak Hydrometeorological Institute, which is commonly used for the long-term monitoring of forest plants [42]. The group of individuals consisted of 20 bilberry plants growing within a forest stand with good health conditions. Visual observations of bilberry shoots were conducted weekly in the same place in each research area (900 m^2^) from 2016 to 2021. The selected phenological phases were determined according to the international BBCH scale [43]. The onset of phenological phases was expressed by the number of days counted from 1 January until the day when the given phenophase started (DOY). The following phenological phases were observed:

BBCH 07—Shoot expansion (Leaves are completely open)—vegetative stage
BBCH 65—Full bloom (Flower petals are open for pollination)—generative stage
BBCH 72—Petal fall (Petals drop, but the calyx and stamen remain)—generative stage
BBCH 86—Blue fruit (Fruit is ripe, sugar content is high)—generative stage
BBCH 92—Autumn leaf colouring (Leaves turn red and drop naturally)—vegetative stage

### 2.5. Statistical Analysis

Statistical analyses were performed in R [44]. The significance of the differences was assessed by ANOVA followed by a post hoc Tukey’s HSD test with a significance level of α = 0.05. The degree of correlation between two variables, phenophase and altitude, was expressed by a Pearson correlation coefficient [45]. The homogeneity of the variance was evaluated using Bartlett’s test. The LMM (linear mixed-effects model) was used to test the effect of elevation, temperature, precipitation, density, and habitat on the onset of selected phenological phases of bilberry. The lmer function from the lme4 package in R was used to run a linear mixed-effects model (LMM). This model was chosen because it can handle both fixed and random effects, allowing for an analysis that accounts for variations at different levels. The specification of random effects within the linear mixed-effects model was articulated in R syntax as follows: (1 + alt + Density_avg | Year):

The term (1 | Year) in the model represents a random intercept. This allows the baseline level of phenophases to change yearly, accounting for factors that might differ between years, like climate changes.

A random slope for altitude (alt) means that the effect of altitude on phenophases can vary each year. For example, altitude might have a more substantial impact in some years due to changing environmental conditions.

Similarly, a random slope for average density (Density_avg) lets the model adjust for how the effect of density on phenophases changes from year to year. This could be due to factors like population dynamics or resource availability differences.

After estimating the random effects for each phenophase, a baseline model was created that included temperature and precipitation from the three months before each phenological phase. Then, an R script was used to find the best combination of fixed predictors and their interactions by comparing models using the Akaike Information Criterion (AIC). The model with the lowest AIC was chosen. If models differed by less than 2 AIC units, they were considered equally good; if they differed by 4–10 units, the difference was more pronounced [46].

In the model descriptions, marginal R^2^ expresses the amount of variance explained by the fixed effects alone, while conditional R^2^ accounts for the proportion of variance explained by both the fixed and random effects.

## 3. Results

### 3.1. The Density of Bilberry

**Figure 4:**
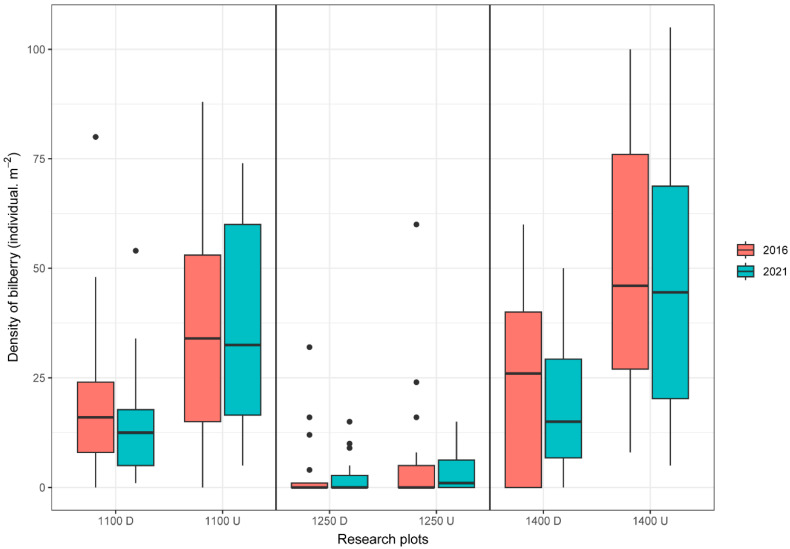
Changes in the density of bilberry shoots in different habitats on a vertical gradient between 2016 and 2021. (U—Undisturbed Forest; D—Disturbed Forest). (Boxplots of median values of temperature and precipitation and corresponding quartiles. Dots in some boxplots represent potential outliers).

The results showed the variability and the differences in the density of bilberry in each research site. However, no significant difference (*p* > 0.05) in the density of bilberry shoots was found on research plots between two years (2016 and 2021). We observed a relatively higher density of bilberry shoots (per m^2^) in 2016 on each control site (localities U) than in 2021. A mutual comparison of undisturbed plots showed the highest density of shoots at 1400 U (49.65 per m^2^), while the lowest density of bilberry shoots was observed at 1250 U (7.4 per m^2^). Similarly, as in undisturbed forests, the density of bilberry shoots between 2016 and 2021 showed no significant difference (*p* > 0.05) on disturbed plots by bark beetles (localities D). The highest density of bilberry shoots was found on plots 1400 D (22.8 per m^2^) and 1100 D (20.6 per m^2^) in 2016, while in 2021, it was 17.9 individuals per m^2^ (1400 D) and 15.05 individuals per m^2^ (1100 D). We found 3.4 individuals per m^2^ on plot 1250 D in 2016, while in 2021, it was 2.75 individuals per m^2^ (19.11% decrease). On the other hand, a significant difference (*p* < 0.05) in the density of shoots was found between undisturbed spruce forests (control plots) and sites disturbed by bark beetles (localities D) in 2016 and 2021 (Figure 4).

### 3.2. Phenology of Bilberry (2016–2021)

The onset of selected phenological phases of bilberry shoot at different habitats in the period 2016–2021 is described in Table 3. As we see, interannual variability in the onset of vegetative phenological phases, as well as generative phenological phases, was determined in all research sites. However, we found that elevation had no significant effect on the average onset of selected phenological phases of the bilberry shoot. On average, the earliest onset of selected phenological phases was found in disturbed forests (localities D). On average, bilberry leafing or onset of shoot expansion (BBCH 07) in the lowermost site (locality 1100 D) was recorded on 127th DOY (i.e., 7 May). However, variation among the dates is considerable between the 25th of April in 2018 (115th DOY) and the 17th of May in 2017 (137th DOY). The onset of BBCH 07 was delayed progressively with increasing altitude; the 7 days (i.e., 14 May) delay was recorded at the highest altitudes in localities D. A similar course was found within the subsequent three phenological phases—BBCH 65, BBCH 72, BBCH 86 when the difference ranged from 5 to 11 days along the altitudinal gradient. On the other hand, on average, the earliest onset of BBCH 92 was observed at the uppermost site (1400 D). It gradually delayes with decreasing altitude. Therefore, the latest onset of BBCH 92 was at the lowest-lying sites (1100 D). Phenological monitoring of bilberry populations from undisturbed spruce forests (localities U) shows a similar trend as found in bilberry populations from disturbed forests (localities D). Phenophase BBCH 07 in the undisturbed forest (site 1100 U) was first recorded in the lowermost site (i.e., on the 128th DOY to 8 May), whereas it was recorded on the 135th DOY (i.e., 15 May) in the uppermost site (localities 1400). The onset of the next phenophases, BBCH 65, BBCH 72, and BBCH 86, had a similar course (with increasing altitude, the onset of the selected phenophase was delayed to later dates). The exception is autumn phenophase (BBCH 92). With increasing altitude, the onset of BBCH 92 phenophase shifted to the earlier dates. The difference in the onset of BBCH 92 between the highest and the lowest altitudinal sites (1400 U–1100 U) was −6.2 days. Furthermore, the average onset of selected phenological phases of bilberry shoots was also not significantly affected by habitats (localities D vs. localities U). The differences between the average onset of phenological phases and habitats (localities D vs. localities U) were minimal. The average onset of BBCH 10 in site 1100 U reached −1.5 days delay with compared 1100 D. A similar course was found within the next phenophase—BBCH 65 (−2.5 days), BBCH 72 (−5.5 days) BBCH 86 (−2.2 days) and BBCH 92 (−3.3 days). On the other hand, a relatively lower delay of average onset of BBCH 10 (−1.3 days), BBCH 65 (−1.7 days), BBCH 72 (−2.2 days), and BBCH 86 (−2.1 days) was found by comparing both sites in 1400 m a.s.l. (research plots D vs. research plots U). Negligible differences in the delay of BBCH 92 phenophase were found between 1400 U and 1400 D. The average duration of the growing season for bilberry, defined as a period between the average onset of BBCH 07 and BBCH 92 phenophases, lasted from 132 days (1400 U) to 150 days (1100 U) depending on the altitude. On average, about 12% shorter growing season was observed at the highest altitudes than the lowest ones. The duration of the growing season at disturbed forests (localities D) had a very similar trend, ranging from 134 days (1400 D) to 149 days (1100 D).

### 3.3. Effects of Environmental Factors in the Phenology

At BBCH 07, the impact of February temperature, precipitation of March, and Altitude were found to be positively significant. On the other hand, March’s temperature and January’s precipitation were found to be negatively significant (Table 4). The impact of other fixed effects (habitat and altitude×habitat) was not significant at (*p* > 0.05). Temperature (between June and September) and precipitation (July and August) were not included in the model because the average onset of BBCH 07 begins in May. The model performed well with a marginal R^2^ of 0.516 and a conditional R^2^ of 0.968. A high marginal R^2^ denoted those fixed effects explained most of the variance in the model.

At BBCH 65, temperature and precipitation after May were not included in the model as they did not significantly correlate with BBCH 65. The temperature of February and altitude × habitat interaction did not substantially affect the BBCH 65 (*p* > 0.05). The marginal R^2^ was higher (0.716), indicating a higher variance was explained by the fixed effects, whereas the conditional R^2^ was 0.977, indicating the overall variance of the fixed and random effects.

At BBCH 72, the impact of all fixed effects was not significant at *p* > 0.05. Marginal R^2^ (0.252) and conditional R^2^ (0.405) were small, indicating a slight variance was explained by the fixed effects.

At BBCH 86, just altitude was a significant variable when comparing all fixed effects. Other fixed effects were found not significant. A marginal R^2^ was also small (0.129), whereas the conditional R^2^ was relatively high 0.917.

At BBCH 92, the impact of August precipitation, habitat, and altitude*habitat interaction was insignificant (*p* > 0.05). The model showed that temperature (July and August), precipitation in July, and altitude are negatively related to BBCH 92 but positively related to the temperature in September (significant effect). The model explained 90% (conditional R^2^ = 0.909) of the variation in BBCH 92.

## 4. Discussion

### 4.1. Characteristics of Bilberry Population Growing in the Different Habitats

The abundance of bilberry shoots and other herb species depends on the forest condition. One of the most important factors influencing the abundance of bilberry in forest floor vegetation is the tree layer [47]. Several studies have described the impact of tree canopy on-air mixing, force evapotranspiration, and absorption radiation in the forest understorey [48,49]. However, ecological results related to the state of the forest and bilberry populations showed that, in the year of analysis (2016) and rechecking year (2021), bilberry plants have a higher density (but not significant at *p* > 0.05) in the undisturbed spruce forest (each control plot) than in the disturbed forest. We agree that crucial factors for bilberry cover (density) are stand density and canopy structure, as [47] suggested. Canopy and stand density in a mountain forest influence other factors, e.g., moderate temperature, light interception, wind and rain protection, precipitation, humidity, soil fertility, and nutrient supply closely related to the herbal layer [50,51,52]. The herb layer in the undisturbed spruce forest was relatively stable during the study period. In general, we found acidophilic species like *Oxalis acetosella*, *Rubus hirtus*, and *Dryopteris dilatata.* The increasing altitude added subalpine plant species, *Homogyne alpina* and *Luzula sylvatica*. Recent studies from the Central European and Scottish forests have shown that bilberry grows best at canopy covers of around 50% [53] in the acidic coniferous forest [47,54]. This finding shows the importance of the forest canopy in regulating light supply into the understory layer [55,56]. So, understory vegetation lives in the shade of a more or less continuous overstory. We suppose that light in closed forests may limit the growth, reproduction, and density of the bilberry population. Moreover, this study shows that bilberries were abundant (density) in sunny sites. These sunny sites were situated in an old open forest (upper part of the hill) with a stocking of 0.6 and also in the young forest (lowest part of the hill) with a stocking of 0.8 (it had small and medium gaps). Thus, the light level should have a similar impact on the bilberry cover in old (165 years) and relatively young forests (100 years). This corresponds with other studies [47,57], which say light is central to bilberry distribution, habitat, or quality. However, in the middle, standing in an undisturbed forest (1250 m a.s.l.), the density of bilberry is very low. These stands were strongly influenced by wind disturbances in 2014. In the year of analysis (2016), only a slight population of bilberry was recorded immediately after the disturbance. It is possible that downed trees change forest structure and function (e.g., increased light intensity, air temperature changes, air humidity decreases), thus changing the microclimate and the present species composition [58,59]. We suppose that these changes in conditions were not ideal for bilberry cover because this acidophilic chamaephyte may not adapt fast enough. For these reasons, the bilberry cover disappears in the middle stand (1250 U). On the other hand, sites where the tree species composition has been strongly affected by bark beetles may increase light availability. The amount of dead wood increases the diversity of herbs and plants [60], fungi [61], and animals [62,63]. In general, the species richness observed in disturbed forests was much higher compared with natural forests. This is consistent with the previous studies [64,65]. Disturbed forests have more fluctuating temperatures [66] and precipitation [67], which is beneficial for pioneer shrubs, trees, and some herb species. In disturbed forests, bilberry cover is situated in the understory of plant species typical for clearings (*Avenula flexuosa*, *Calamagrostis villosa*, *Chamaerion angustifolium*, *Luzula luzuloides,* and *Rubus idaeus*). These sites are relatively shady and have sufficient humidity and a lower pH (typically between 4.0 and 5.5), which is necessary for the growth of the bilberry population. Our study also observed bilberry cover in small niches around dead-standing trees or dead stumps. We suppose that these sites prevented the penetration of plant species with high light and heat requirements. This means that these niches create good conditions for the reproduction of bilberry.

### 4.2. Phenology of Bilberry Population Growing in the Different Habitats

The main objective of our study was to determine the interannual dynamics of phenological events in bilberry along the elevational gradient in different habitats. Although the phenological observation of the bilberry population was short (2016–2021) and insufficient in the context of climate change, an intriguing pattern of elevation influences was demonstrated. The LMM analysis pointed to the clear influence of elevation and habitat on bilberry phenology (Table 4). As expected, an overall shift in bilberry phenology towards later occurrences and shorter growing seasons was observed with increasing elevation. In addition, phenology development and the onset of selected phenophases start earlier in disturbed forests. This finding aligns with the hypothesis that warmer temperatures and increased light availability in disturbed areas accelerate plant development. However, the differences in the onset of selected phenophases in different habitats at the same altitude were minimal. For example, the average onset of BBCH 07 in undisturbed spruce forests reached a 1.5-day delay compared to the disturbed forest at 1100 m a.s.l (Table 3). A similar course was found at 1250 m a.s.l. (+1 day) and at 1400 m a.s.l. (+1.3 days). The average onset of another reproductive stage (BBCH 65, BBCH 72, and BBCH 86) in bilberry shows higher variability. However, the average onset of the last observed vegetative phenophase (BBCH 92) in undisturbed spruce forests reached +3.3 days (1100 m a.s.l.), +1.2 days (1250 m a.s.l.) and 0 days (1400 m a.s.l.) of delay compared to the disturbed forests. We assume that differences in local abiotic factors (temperature, water, and nutrient availability) and biotic interactions (bark beetle outbreaks) were not significant enough to cause a different onset of selected phenophases in various habitats along an altitude gradient. The most important factor affecting bilberry phenology on the elevation gradient is the temperature regime during the growing period [68,69,70,71,72,73]. Temperature emerged as a critical factor affecting the timing of bilberry phenology, as demonstrated by the LMM analysis (Table 4). Our models revealed that warmer temperatures at lower elevations, particularly in disturbed forest stands, were associated with an earlier onset of key phenological events. The beginning of phenological activity may also be affected by the late winter period (snow depth, strong frosts) and the early spring (snowmelt, late frosts). Our research shows that bilberry populations are well adapted to overwintering under snow cover, which lasts more than 5 months on average at 1000 m a.s.l. in Tatra Mts. (from November to the end of March). During the wintertime, bilberries have an insulating snow cover, which protects the bilberry shrubs and provides the plant with the moisture it needs long into the spring. Stable snow cover and stable temperatures in winter are important for bilberry populations because they are sensitive to frosts [69,74]. It was assumed that the development of bilberry starts after snowmelt [68,71]. However, we found that low temperatures and frosts create problems for the bilberry populations along the altitudinal gradient after snowmelt (see Table 2). We recorded low temperatures in April (2017 and 2021 years), May (2019, 2020, and 2021 years), and also some frosty days in May (2016 year). This period (April and May) is important for bilberry’s physiological and phenological development (e.g., leafing and flowering). In contrast, relatively higher spring temperatures in April (2018 and 2019) and May (2016, 2017, and 2018) without late spring and summer frosts caused earlier floral development of bilberry because of less snow cover. Therefore, we recorded the shifting of selected phenophases to earlier dates. This also affected the duration of the growing season in these years, which was the longest on average. It could foster a bilberry fitness population by extending the period available for growth and resource allocation. However, the bilberry population is relatively frost-sensitive during the increasing spring temperatures (in the April–May period), when frost damage to the bilberry population strongly increases. On the 17th of May 2016, frosts damaged the flowers in the bilberry population. The withered, unopened crown petals prevented bee pollination, which may indirectly affect fruit and seed quantity and quality [75,76]). On the other hand, frost-damaged flowers drop rather than develop into fruit [71] and severely reduce plant growth [77]. This phenomenon affected populations of bilberry in disturbed forests at 1400 m a.s.l. We assume that exposed sites (mostly damaged forests) decreased protection from low spring temperatures because the tree layer did not provide a protective shield. However, [71] reported that bilberry populations can generally replace aborted shoots and leaves in the same growing season. Similar statements were published by [78], who stated that the bilberry could recover from shoot damage caused by frost by mobilising stored nutrients into regenerative growth. For this reason, we assumed that the availability of nutrients would decrease with higher elevations, and it would be the limit for the growth and reproductive phenology of bilberry [32]. A 15-year investigation from Sweden confirmed the same findings [79]. They concluded that spring frost during flowering caused low or zero berry production.

## 5. Conclusions

We analysed the bilberry population in natural mountain spruce forest stands affected by windstorms in 2004 and 2014 and consequently disturbed by bark beetles. Based on our research, we can conclude that the type of habitat and elevation are the factors that affect the density and phenology of bilberry. A significantly higher density of bilberry shoots was observed in undisturbed spruce forest stands than in disturbed forest stands along the entire altitudinal gradient (1100–1250–1400 m a.s.l.). However, no significant differences were found in the density of bilberry shoots over time (compared to the 2016 and 2021 years). The results of phenological observations showed that habitat and altitude affected the onset of selected phenological phases. The LMM showed significant interactions between altitude and habitat type, suggesting that the phenological development of bilberry is not solely driven by temperature gradients but also by local ecological conditions, such as soil properties and microclimatic variations induced by forest structure. The earliest onset of selected phenophases of bilberry was found at low altitudes (1100 m a.s.l.) in disturbed forest stands. Conversely, the latest onset of the phenophases was observed at the highest altitudes (1400 m a.s.l.) in the undisturbed forest stand. These results confirm that bilberry (*Vaccinium myrtillus* L.) can partially grow in disturbed forest stands and profit from a longer growing season despite the temperature fluctuation during the early spring period (early and late frost). This resilience indicates that bilberry can adapt to different environmental conditions, but the long-term effects of these disturbances on bilberry populations require further study.

## Figures and Tables

**Table 1:** Description of the studied sites—undisturbed forest spruce stands (U) and disturbed forests by bark beetles (D) along the vertical transect.

Habitat	Plot	Alt (m a.s.l.)	WGS (N, E)	Soil	Surface	Slope (°)	Exp	Age (Years)	Stocking
Undisturbed	1100 U	1100	49°10′29.33′ N, 20°14′45.12′ E	Dystric Cambisol	Stony	10	SE	100	0.8
Undisturbed	1250 U	1250	49°10′36.91′ N, 20°14′32.59′ E	Dystric Cambisol	Stony	35	SE	165	0.4
Undisturbed	1400 U	1400	49°10′51.03′ N, 20°14′24.14′ E	Podzol	Boulder	35	SE	165	0.4
Disturbed	1100 D	1100	49°10′28.03′ N, 20°14′43.08′ E	Dystric Cambisol	Stony	10	SE	-	-
Disturbed	1250 D	1250	49°10′34.59′ N, 20°14′31.05′ E	Dystric Cambisol	Stony	35	SE	-	-
Disturbed	1400 D	1400	49°10′52.82′ N, 20°14′26.67′ E	Podzol	Boulder	35	SE	-	-

**Table 2:** Mean temperature along the vertical gradient during the study period.

	Mean Air Temperature of April (°C)			Mean Air Temperature of May (°C)			Mean Air Temperature of GS * (°C)			Mean Annual Air Temperature (°C)		
Altitude (m a.s.l.)	1100	1250	1400	1100	1250	1400	1100	1250	1400	1100	1250	1400
2016	5.7	4.9	4.1	9.6	8.6	7.6	11.5	9.6	9.9	5.4	4.8	4.1
2017	3.4	2.5	1.5	10.1	9.2	8.4	11.2	10.3	9.5	5.6	5.3	4.9
2018	9.9	9.0	8.2	12.3	11.5	10.6	13.0	11.4	11.3	6.1	5.5	4.9
2019	5.7	4.8	3.8	7.5	6.6	5.7	11.5	10.3	10.0	6.2	5.6	5.1
2020	5.3	4.4	3.6	7.1	6.1	5.1	11.1	9.6	9.5	5.9	5.4	4.9
2021	1.5	0.6	−0.3	7.6	6.6	5.7	10.6	9.0	8.9	4.8	4.2	3.6
Mean	5.3	4.4	3.5	9.0	8.1	7.2	11.5	10.0	9.6	5.7	5.1	4.6

* GS = Growing season

**Table 3:** The onset of selected phenophases of bilberry during 2016–2021. Mean ± standard deviations are displayed.

	BBCH 07	BBCH 65	BBCH 72	BBCH 86	BBCH 92
1100 D	127 ± 8.80	143 ± 10.50	153 ± 13.52	232 ± 7.26	276 ± 10.34
1250 D	130 ± 8.90	147 ± 10.09	154 ± 9.69	234 ± 6.86	274 ± 10.93
1400 D	134 ± 10.63	154 ± 8.24	160 ± 7.90	237 ± 4.99	268 ± 12.75
1100 U	128 ± 8.65	146 ± 10.28	158 ± 11.72	234 ± 5.98	278 ± 12.75
1250 U	130 ± 8.28	148 ± 10.14	159 ± 9.62	235 ± 6.50	275 ± 12.78
1400 U	135 ± 10.30	155 ± 8.68	162 ± 7.60	239 ± 4.22	268 ± 11.30

**Table 4:** Linear mixed-effects model for the onset of the selected phenological phases of bilberry from 2016–2021 in the study area. Estimates are derived from the LMM analysis (T2—temperature of February, T3—temperature of March, T4—temperature of April, T5—temperature of May, T6—temperature of June, T7—temperature of July, T8—temperature of August, T9—temperature of September; P1—precipitation of January, P2—precipitation of February, P3—precipitation of March, P4—precipitation of April, P7—precipitation of July; P8—precipitation in August; alt—altitude; R^2^_M_ and R^2^_C_ are marginal and conditional R^2^; Significance codes: * *p* < 0.05, ** *p* < 0.01, *** *p* < 0.001).

	Best Model				
	BBCH 07	BBCH 65	BBCH 72	BBCH 86	BBCH 92
Intercept	110.48 ***	72.05 ***	128.29 ***	236.14 ***	353.28 ***
T2	6.8 **	−1.61			
T3	−5.88 *	6.61 ***			
T6				−1.89	
T7				0.8	−6.14 **
T8					−5.46 *
T9					13.08 ***
P1	−0.54 **				
P2	0.05	0.29 ***	0.1		
P3	0.60 *	0.77 ***	−0.07		
P4			0.05		
P7				−0.04	−0.16 **
P8					0.06
alt	0.02 ***	0.02 ***	0.02	0.02 *	−0.02 *
Habitat [U]	3.5	−5.57 **	0.38	3.71	−0.44
R^2^_M_	0.516	0.716	0.252	0.129	0.829
R^2^_C_	0.968	0.977	0.405	0.917	0.909

## Data Availability

The data presented in this study are available on request from the corresponding author.
